# Supplementary material for: Spatiotemporal dynamics of multi-vesicular release is determined by heterogeneity of release sites within central synapses
Source: eLife. 2020 Feb 6;9:e55210. doi: 10.7554/eLife.55210 (PMC7060041; doi:10.7554/eLife.55210)
Supplement: Transparent reporting form [file elife-55210-transrepform.docx]

***eLife’s*transparent reporting form**

We encourage authors to provide detailed information *within their submission* to facilitate the interpretation and replication of experiments.Authors can upload supporting documentation to indicate the use of appropriate reporting guidelines for health-related research (see [EQUATOR Network](http://www.equator-network.org/%20)), life science research (see the [BioSharing Information Resource](https://biosharing.org/)), or the [ARRIVE guidelines](http://www.plosbiology.org/article/info:doi/10.1371/journal.pbio.1000412) for reporting work involving animal research.Where applicable, authors should refer to any relevant reporting standards documents in this form.

If you have any questions, please consult our Journal Policiesand/or contact us:[editorial@elifesciences.org](mailto:editorial@elifesciences.org).

**Sample-size estimation**

- You should state whether an appropriate sample size was computed when the study was being designed
- You shouldstate the statistical method of sample size computation and any required assumptions
- If no explicit power analysis was used, you should describe how you decidedwhat sample (replicate) size (number) to use

Please outline where this information can be found within the submission (e.g., sections or figure legends), or explain why this information doesn’t apply to your submission:

We followed an exploratory research approach, and we did not have an initial estimate for the type of analysis, results, or errors we would observe for each experiment. Thus, we could not perform an initial power analysis for this work. However, we did estimate the number of experiments we would need based on our previous published works (Maschi and Klyachko 2017, Maschi et al., 2018). Both studies suggested a minimum of 3 independent cultures and 9 coverslips per experiment.

**Replicates**

- You should report how often each experiment was performed
- You should includea definition of biological versus technical replication
- The data obtained should be provided and sufficient information should be provided to indicate the number of independent biological and/or technical replicates
- If you encountered any outliers, you should describe how these were handled
- Criteria for exclusion/inclusion of data should be clearly stated
- High-throughput sequence data should be uploaded before submission, with a private link for reviewers provided (these are available from both GEO and ArrayExpress)

Please outline where this information can be found within the submission (e.g., sections or figure legends), or explain why this information doesn’t apply to your submission:

All information about the number of replicated experiments, biological samples, and data obtained is listed in Table 1. Information is listed for each condition and associated with each figure where the data is presented. Information about data exclusion criteria is listed in Methods.

**Statistical reporting**

- Statistical analysis methods should be described and justified
- Raw data should be presented in figures whenever informative to do so (typically when N per group is less than 10)
- For each experiment,you should identify the statistical tests used, exact values of N, definitions of center, methods of multiple test correction, and dispersion and precision measures (e.g., mean, median, SD, SEM, confidence intervals; and, for the major substantive results, a measure of effect size (e.g., Pearson's r, Cohen's d)
- Report exact p-values wherever possible alongside the summary statistics and 95% confidence intervals. These should be reported for all key questions and not only when the p-value is less than 0.05.

Please outline where this information can be found within the submission (e.g., sections or figure legends), or explain why this information doesn’t apply to your submission:

All statistical tests used are listed in Figure legends and Methods Section. All N-values are listed in Figure legends and Table 1. All p-values are listed in Table 1. All calculations of means and SEM are listed in Table 1. All p-values above 0.001 are stated as exact values.

(For large datasets, or papers with a very large number of statistical tests, you may upload a single table file with tests, Ns, etc., with reference to sections in the manuscript.)

**Group allocation**

- Indicate how samples were allocated into experimental groups (in the case of clinical studies, please specify allocation to treatment method);if randomization was used, please also state if restricted randomization was applied
- Indicate if masking was used during group allocation, data collection and/or data analysis

Please outline where this information can be found within the submission (e.g., sections or figure legends), or explain why this information doesn’t apply to your submission:

All information about group allocation is listed in the Methods section.

**Additional data files (“source data”)**

- We encourage you to upload relevant additional data files, such as numerical data that are represented as a graph in a figure, or as a summary table
- Where provided, these should be in the most useful format, and they can be uploaded as “Source data” files linked to a main figure or table
- Include model definition files including the full list of parameters used
- Include code used for data analysis (e.g., R, MatLab)
- Avoid stating that data files are “available upon request”

Please indicate the figures or tables for which source data files have been provided:

There are no additional data files.
